# Comparing oxygen demand in critical covid-19 patients using single versus double doses of tocilizumab

**DOI:** 10.15537/smj.2023.44.5.20220755

**Published:** 2023-05

**Authors:** Abdullah U. Althemery, Marzoog A. Albadi, Ahmad F. Allaf, Shekhah S. Almoqren, Amal H. Alnajjar, Faisal K. Alkholifi

**Affiliations:** *From the Department of Clinical Pharmacy (Abdullah, Fisal), College of Pharmacy, Prince Sattam bin Abdulaziz University, Al-Kharj; from the Internal Medicine Department (Marzoog, Ahmad) and Pharmacy Department (Amal), Security Forces Hospital; and from Pharmaceutical Care Services (Shekhah), King Abdulaziz Medical City, Ministry of National Guard Health Affairs, Riyadh, Kingdom of Saudi Arabia.*

**Keywords:** COVID-19, tocilizumab, double dose, Saudi Arabia, antiviral, oxygen demand

## Abstract

**Objectives:**

To compare the outcomes of single versus double doses of tocilizumab in patients with severe COVID-19, especially on different types of oxygenation requirements.

**Methods:**

This cross-sectional study was carried out from January 2020 to March 2020. Patients diagnosed with COVID-19, who received at least one dose of tocilizumab, were included. The dependent variable was tocilizumab dose (single versus double). The primary outcome variable was oxygen demand on the first and last day of hospitalization. A series of comparisons between patients administered one dose of tocilizumab versus 2 doses were conducted.

**Results:**

Herein, 80 patients with severe COVID-19 infection were included, of whom 68.8% received one dose of tocilizumab, while 31.3% received a double dose. Two-thirds of the patients were male, with an overall average age of 58 years. In patients receiving 2 doses, oxygen demand tended to worsen by the seventh day, while in those who received one dose. The group that received 2 doses had a longer length of hospital stay.

**Conclusion:**

This study could not capture the additional value of the second dose for different health outcomes. However, the results can inform clinician from experience when facing uncertainty due to new virus or variant.

**A**t the end of 2019, the world faced a health emergency due to the coronavirus disease 2019 (COVID-19) outbreak. It began in China, spread across the globe, and was declared a global health crisis.^1^ COVID-19- associated respiratory failure became one of the main causes of death in January 2020 and remained so until the end of 2021.^2^ COVID-19 patients, particularly those with severe and critical comorbid conditions, were the most affected.^3^

Observational studies from Saudi Arabia examining the effectiveness of tocilizumab in patients with COVID-19 were inconclusive.^4,5^ Al-Baadani et al^4^ reported that critically-ill patients treated with tocilizumab show a lower mortality rate than a control group receiving standard care. In contrast, AlQahtani et al^5^ suggested no association between tocilizumab administration and mortality among patients with severe-to-critical COVID-19 infection.^5^ A similar trend has been observed in international reports evaluating tocilizumab and COVID-19-associated mortality.^6^

Few studies have focused on evaluating the effects of tocilizumab dosing (single versus multiple doses) on the outcomes of patients with COVID-19.^7,8^ All of them have focused on mortality and the need for mechanical ventilation but have overlooked the effects of tocilizumab on oxygen demand across different oxygen delivery types. Thus, the goal of this study was to assess the short-term effects of single versus double doses of tocilizumab on outcomes (including different types of oxygenation requirements) in patients with severe COVID-19 infection.

## Methods

This cross-section was carried out from January 2020 to March 2020, in the Drug and Poison Information Center (DPIC) at the Security Forces Hospital (SFH), Riyadh, Saudi Arabia. It is a tertiary hospital with over 500 beds that offers healthcare for the Ministry of Interior employees and their families. The Institutional Review Board (IRB) approval was obtained from the research committee of SFH (IRB number H-01-R-069).

All study participants were followed up from one day after admission until the day of discharge or death. The study sample included patients diagnosed with COVID-19 who received at least one dose of tocilizumab. The diagnosis of COVID-19 was confirmed twice by reverse transcriptase-polymerase chain reaction (RT-PCR) using swab samples obtained from the nasopharynx or throat. Tocilizumab dosing regimens were based on the MoH-KSA protocols and SFH internal guidelines.^9^ Patients with less than a day of admission, who previously received tocilizumab, or who had a do-not-resuscitate order were excluded. The study data were collected from the patients’ medical records. The dependent variable was the dose of tocilizumab and was categorized as single versus double. The primary outcome variable was oxygen demand on the first and seventh day of hospitalization. The latest oxygen demand was recorded if patients were discharged or died before the seventh day. Oxygen demand was classified into 4 following groups (or scores): 1- room air with (score 0), 2- low flow and moderate with an airflow up to 15 L (score 1); 3- high flow more than 15 L; and either through high flow nasal cannula or noninvasive ventilation (score 2), and 4-mechanical ventilation (score 3). Other secondary outcomes included the duration of hospital stay, and survival. The independent variables were classified into the following 4 groups: categorical data for patient characteristics (sex, diabetes, hypertension, cardiovascular disease, asthma, chronic kidney disease, chronic obstructive pulmonary disease, and hepatitis), continuous data on patient characteristics (age, temperature, respiratory rate, heart rate, hemoglobin, white blood cells, and lymphocytes), COVID-19 signs and symptoms (fever, shortness of breath, cough, contact with COVID, sore throat, diarrhea, loss of taste, and loss of smell), and therapy (anticoagulants, antiviral, corticosteroids, and hydroxychloroquine).

### Statistical analysis

A series of comparisons between patients administered one dose of tocilizumab versus two doses were conducted using the Chi-square test, Fisher’s exact test, and t-tests with a significance level of 0.05. The association between tocilizumab and the primary outcome, such as, oxygen demand on days 1 and 7, was assessed by analysis of variance (ANOVA) for differences with a significance level of 0.05. Moreover, a sensitivity analysis was conducted for the primary outcome, whereby oxygen demand was tested using a non-parametric test, Wilcoxon’s signed-rank method. Another sensitivity analysis was conducted to address the limited sample size, where bootstrapping technique applied with 1000 replicate drawn from the original sample; bootstrapping is a computerized method addresses estimate uncertainty in data with limited samples by producing new experiments from simulated samples. The ordinal logistic regression analysis was used for the bootstrapped sample to estimate the oxygen demand at the 7th day (main dependent variable) from tocilizumab dosing (main independent variable). All statistical tests were performed, and graphs were plotted using Microsoft Excel and R: A language and environment for statistical computing.

## Results

A total of 80 patients with severe COVID-19 infection were included in the study, of whom 68.8% received one dose of tocilizumab, while 31.25% received 2 doses. The majority of the patients were men (73.8%), with an overall average age of 58 years (SD=13.88). In terms of medical conditions, most patients had no history of major chronic conditions, such as diabetes (48.8%), hypertension (46.3%), cardiovascular diseases (11.3%), asthma (11.3%), chronic kidney diseases (11.3%), chronic obstructive pulmonary diseases (2.5%), or hepatitis (1.3%).

The oxygen demand during the first day of hospitalization was comparable between the 2 groups. More patients had low-flow oxygen (66%), followed by high-flow (27%), and mechanical ventilation (6%). However, the relationship between oxygen demand on the first day and tocilizumab use was statistically insignificant. Similarly, on the seventh day of hospitalization, more patients required low-flow ventilation (33%). However, one-third of all patients required mechanical ventilation after a week (30%), and the percentage was higher in patients who received double doses (36%) than the single dose (26%). Thus, the relationship between oxygen demand after a week and tocilizumab therapy was statistically significant (X2 (3, n=80)=9, *p*=0.02).

**Figure 1:**
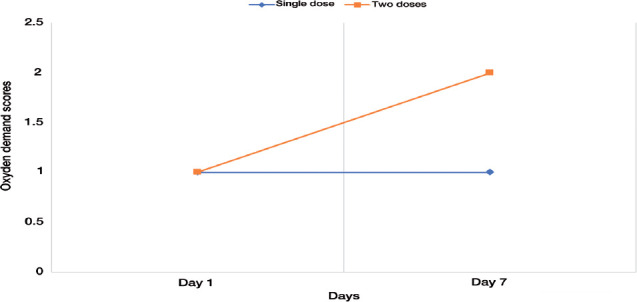
- Median oxygen demand on days 1 and 7.

For the primary outcome variable, such as oxygen demand, the data showed that patients who were administered 2 doses tended to worsen by the seventh day (Figure 1), while those receiving one dose showed no improvement; the median number of patients receiving low-flow oxygen on day number 1 and 7. The ANOVA for the difference in oxygen demand between pre- and post-test yielded statistically significant differences between the first and seventh day within the 2 groups, F (2, 80)=17, *p*=0.01.

**Figure 2:**
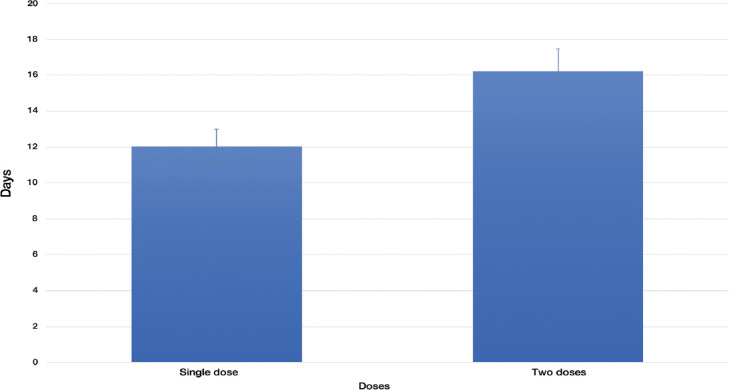
- Comparison of mean hospital length of stay.

**Figure 3:**
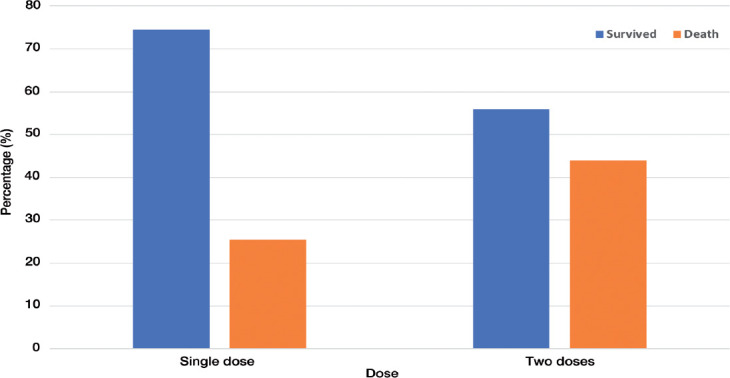
- Comparison of survival rates.

Hospital length of stay (LOS) was compared between the 2 groups of patients (Figure 2). There was a statistically significant difference between the 2 groups, and the 2 doses group was associated with a higher LOS (M=12.05±6.83) relative to the one dose group (M=16.20±6.36) of tocilizumab, t (80)=2.57, *p*=0.01. There were no significant differences between the 2 groups in terms of average survival (Figure 3).

Sensitivity analysis was conducted to estimate the impact of tocilizumab dosing on the primary outcome (oxygen demand) using ordinal logistic regression from bootstrapped sample; the final model showed that the utilization of the second dose of tocilizumab decreased oxygen demand (the progression of patients from “room air” to “low flow and moderate airflow”, from “low flow and moderate airflow” to “high flow, or nasal cannula or noninvasive ventilation”, and from “high flow, or nasal cannula or noninvasive ventilation” to “mechanical ventilation”). However, the estimate of tocilizumab was not significant.

## Discussion

This study aimed to evaluate the differences in health outcomes between patients receiving one dose of tocilizumab and those receiving double doses. Overall, there were no significant differences between the 2 groups in baseline characteristics. These results were consistent with local and international studies.^7,9^ For instance, Al Sulaiman et al^8^ showed that an increased dose of tocilizumab did not improve the mortality rate in severely-ill COVID-19 patients. Similarly, Durán-Méndez et al^10^ found a limited benefit when higher doses of tocilizumab were administered in the early stages of COVID-19.

There was a significant relationship between oxygen demand and tocilizumab dosing, as the second dose was associated with higher oxygen demand. This finding was confirmed by a sensitivity analysis. This could have resulted from the fact that some treating physicians found no other treatment to resort to, and attempted to improve patients’ clinical status even if evidence of benefit was theoretical; this phenomenon has been observed worldwide throughout the pandemic.^11^

Another significant finding was that the length of hospital stay was, on average, for a single dose less than 2 weeks. Studies from China showed that the majority of patients had LOS of 14 days in the hospital, while in our cohort average LOS was 12 days.^12^ These results may confirm the significance of one dose of tocilizumab in reducing the length of hospital stay, which is consistent with previous findings.^13^ However, our main analysis was conducted to establish the benefits of the second dose, and the results suggest otherwise.

### Study limitations

It was a cross-sectional study that is reflective of the experience at one center. Through the course of the pandemic, the virus has mutated and the death rates have decreased which could be attributed to an increased vaccination rate and better clinical management.^8^ This study was also carried out at a time when the literature on COVID-19 was rapidly changing, as were treatment guidelines, which may have led to a discrepancy in the treatment of patients enrolled at the beginning versus at the end of the study period. Another limitation was that the measurement of oxygen demand was categorized into 4 groups and can be extended with larger sample size.

To address some of these limitations, this research adapted bootstrapping sampling technique which produces unbiased estimate driven from resampled data. Which allowed the distribution of the data to adapt ordinal logistic regression, yet the results remained unchanged were the dose of tocilizumab did not significantly predict the demand of oxygen. However, notably the relationship differ where patients with double doses would require less invasive oxygen demand. Future studies should examine the benefits of additional treatments with a larger number of patients, to determine their effect on oxygen demand.

In conclusion, this study could not capture the value of the second dose for different health outcomes, including ventilation demand, length of hospital stay, and survival. This shown a degree of uncertainty when facing new infection that resulted in various treatment methods, however, a lot could be learned from these experiences to improve the preparedness for the future. Also, the clinical guidelines for tocilizumab use should be reformed to improve the recommendations for the use of the second dose and the time of administration so that patients benefit more.
